# Acknowledgment to Reviewers of *Membranes* in 2021

**DOI:** 10.3390/membranes12020155

**Published:** 2022-01-27

**Authors:** Membranes Editorial Office

**Affiliations:** MDPI AG, St. Alban-Anlage 66, 4052 Basel, Switzerland

Rigorous peer-reviews are the basis of high-quality academic publishing. Thanks to the great efforts of our reviewers, *Membranes* was able to maintain its standards for the high quality of its published papers. Thanks to the contribution of our reviewers, in 2021, the median time to first decision was 13 days and the median time to publication was 31 days. The editors would like to extend their gratitude and recognition to the following reviewers for their precious time and dedication, regardless of whether the papers they reviewed were finally published:
Abdala, AhmedLi, JianAbdo, HanyLi, JieAbdul-Hamid, Muhammad AzmiLi, JunshengAbdur, RahimLi, LinAbeynayaka, AmilaLi, PeiAbliz, AblatLi, PengAboraia, AbdelazizLi, WanbinAcero, Juan L.Li, YuanchaoAchiou, BrahimLi, ZhiliAcquah, SteveLi, ZhipengAdams, DavidLian, ‪BoyueAfsari, MortezaLiang, Can ZengAguilar-Bolados, HéctorLiang, XueleiAguilella-Arzo, MarcelLiao, Shu-ChuanAhmad, Mohd ZamidiLiao, Wei-ChingAhmadian, AliLim, SoomanAhmed, FaizLim, SungilAhn, EungjinLin, Chien-hongAhn, Jun KiLin, HaiqingAkhtar Qaisrani, NaeemLin, HonghongAkhtar, FaheemLin, Yu-TingAkhtar, Faheem HassanLindemann, StephanAkimov, Sergey A.Liu, GuichengAkutsu, KazuhiroLiu, JialeiAl Hseinat, EmadLiu, JunyiAl Munsur, Abu ZafarLiu, RuilinAlaimo, AlessandroLiu, XiaominAlam, Arif UlLiu, YaodongAlapan, YunusLlosa Tanco, Margot A.Alcântara Pereira, Luiz AntonioLong, Nguyen Van DucAlcaraz, LorenaLonghini, FedericoAlfè, MichelaLontio Fomekong, RoussinAlgieri, CatiaLopes, Coeli M.B.Ali, GomaaLopez Martinez, Maria JoséAli, ImranLópez, JulioAli, KhatibiLópez-Zavala, RicardoAli, Syed AzmalLoPresti, PatriziaAliyu, AliyuLoulergue, PatrickAlsalhy, QusayLousada-Ferreira, MariaAlsohaimi, IbrahimLow, NicholasAltosaar, IllimarLoza, NataliaAlvarez-Malmagro, JuliaLunter, DominiqueAnbazhagan, SathiyaseelanLuo, HongxiAnca, FilimonLupo, DonaldAndersson, JakobLuque, SusanaAnnesini, Maria CristinaMa, CanghaiAntunes, RodrigoMa, Chih-MingAnwar, FarhanaMa, GuanyuArafat, HassanMa, Lay SunArellanes-Robledo, JaimeMagdalena, Musuc AdinaAriani, AlaricoMahandra, HarshitAriga, KatsuhikoMahmoodi, NiyazArkas, MichaelMahmoud, Alaa El DinÁrki, PálMaitarad, PhornphimonArnusch, Christopher J.Majee, ArghyaArrigoni, CristinaMakisha, NikolayArufe, María CMaleki, RezaArunkumar, AbhiramMalfertheiner, Maximilian V.Ashfaq, MohammadMali, SuzanaAshour, Mohamed LotfyMaliszewska, Irena H.Ashrafi, AmirmansoorMamaliga, IoanAshrafuzzaman, MdMandal, RonitAshtiani, SaeedMangino, GiorgioAsubar, Joel T.Maniti, OféliaAtanasova, Gabriela LachezarovaManning, Gerald S.Atilgan, AhmetManohar, MurliAubert-Pouëssel, AnneManrique Moreno, MarcelaAuvynet, ConstanceMao, KangAvis, ChristopheMarais, StephaneAyyavoo, JayalakshmiMaraqa, Munjed A.Azar, MassoodMarcos, MarcosAzhar, Muhammad RizwanMariasiu, FlorinBabatunde Alayande, AbayomiMarini, Herbert RyanBąchor, RemigiuszMarszałek, JoannaBachurski, DanielMartínez Barrera, GonzaloBae, Byung-ChanMartino, MarcoBai, HongweiMartins, Rui C.Baig, NadeemMartucci, GennaroBairwa, GauravMartyushev, Dmitriy AleksandrovichBalan, AdrianaMarzorati, StefaniaBaldelli, AlbertoMashentseva, Anastassiya A.Barai, Hasi RaniMateo Fernandez, SaraBarbeau, BenoîtMateus, MaríliaBarbosa De Lima, Antonio GilsonMateusz, KorzecBarbosa, ArmenioMáthé, CsabaBardag-Gorce, FawziaMatsubara, KeiichiBarille, RegisMatsumoto, HidetoshiBârsan, NarcisMatsuura, KazunoriBaruah, Ratul Kr.Matsuura, TakeshiBasak, IndranilMatwijczuk, ArkadiuszBasiak, EwelinaMauriz, ElbaBassin, Joao PauloMaveyraud, LaurentBassyouni, MohamedMažeikienė, AušraBattiato, AlfioMazinani, SaeedBawani, ShowkatMazziotti Di Celso, GiuseppeBayer, IlkerMcCartney, FionaBecher, Peter MoritzMcdonald, PeterBeckingham, BryanMehmood, KhalidBehera, KartikMehrizi, Abbasali AboueiBell, Carl-MartinMeiramkulova, KulyashBella, FedericoMeleleo, Daniela AddolorataBellanger, GilbertMelián-Martel, NoemiBelosludtsev, AlexandrMelin, FredericBelosludtsev, KonstantinMelnikov, PavelBelosludtseva, NataliaMelnikov, StanislavBender, JohannesMendiguchía, CarolinaBen-Dov, Iddo Z.Menéndez, MiguelBenedetti, Francesco M.Menger, Fredric M.Benneker, AnneMercante, LuizaBen-Nissan, GiliMerkel, ArthurBera, Oskar J.Mesaros, AmaliaBerezin, AlexanderMestdagh, HélèneBerlina, Anna N.Mezzetta, AndreaBernal, EvaMiao, YuBernardi, SaraMikhelson, KonstantinBhatti, M.MMilovanovic, DragomirBianchi, BiagioMin, JingchunBiondi, BarbaraMiranda, AdelaideBiswas, Manik ChandraMiszczyk, AndrzejBita, BogdanMitko, KrzysztofBizon, NicuMitkowski, PiotrBlandin, GaetanMitra, SomenathBobrova, LudmillaMitu, BogdanaBolbasov, EvgeniyMizsei, RékaBoldura, Oana-MariaMizsey, PeterBonomini, MarioMizuno, ShiroBorderon, CarolineModestov, Alexander D.BOSKO, María LauraModi, AkshayBoulbitch, AlexeiModi, NisargBouley, ReneeMohamed, Hamdy F. M.Bouyer, DenisMohamed, Salah A.Bozso, Sabin J.Mohammadehtisham, KhanBravo, Susana B.Mojiri, AminBreite, DanielMondal, SayanBrioni, MatteoMondal, SudipBritton, JonathanMontone, Carmela MariaBroer, StefanMoon, Seung-HyeonBrogaard Larsen, JulieMoon, Su-YoungBruijn, Johannes DeMoorhouse, AndrewBruno, RosariaMorelli, SabrinaBryniarski, KrzysztofMorelos-Gómez, AarónBuekenhoudt, AnitaMoreno, CésarBulgariu, LauraMoreno, Maria JoãoBunea, Claudiu IoanMorganti, PierfrancescoBuonomenna, Maria GiovannaMorretta, ElvaBurchacka, EwaMotuzas, JuliusBurckhardt, ChristophMousavi, Seyyed MojtabaBusana, MattiaMoutloali, RichardCaldas, PauloMoya, Antonio A.Calvo, José IgnacioMoyseowicz, AdamCamba, A. TundidorMpupa, AneleCamblor, Miguel A.Mrozik, AgnieszkaCameron, DavidMukherjee, MaitreyeeCanetta, ElisabettaMunteanu, Florentina-DanielaCannilla, CatiaMurcia, M.D.Cantore, StefaniaMusante, IlariaCao, BingMushi, Ngesa EzekielCao, HongbinMuster-Slawitsch, BettinaCao, ZishuMuthumareeswaran, M.R.Cappelli, GianniNagata, KojiCăprărescu, SimonaNagy, EndreCaputo, GregoryNaik, Keerti M.Carabineiro, SóniaNaji, OsamahCarmela, ConidiNam, InhoCarmine, ColucciniNapolitano, FilomenaCarneiro, Sueli Coelho Da SilvaNatale, PaoloCarraro, MauroNava, SaraCarullo, GabrieleNeacsu, Ionela AndreeaCarvalho, Marta S.NECHIFOR, Aurelia CristinaCaseli, LucianoNechifor, GheorgheCassano, AlfredoNees, MatthiasCastiglione, KathrinNemes, OvidiuCastro Dominguez, BernardoNemestóthy, NándorCastro, LuísaNeophytou, Christiana M.Castro-Dominguez, BernardoNesterenko, AlexeyCazacu, AnaNesterkina, MariiaCea Mingueza, PilarNg, Ding-QuanCecconet, DanieleNg, Law YongCena, CíceroNguyen, Van-ChucCeran, BartoszNikitas, NikitasCha, Jin-HyeokNikolaeva, DariaChae, Sung HoNinan, NeethuChakravorty, DavidNinomiya, SatoshiChamati, HassanNistor, Ileana DenisaChandra Rao, Varikuti PurnaNogueira, Gislaine FerreiraChang, Hai-ChouNoori, Md TabishChang, Han-WeiNorton, RayChang, Wen-WeiNuez, IgnacioCharfi, AmineNunna, Bharath BabuChawla, PrinceNyamutswa, Lavern T.CH-Chaoui, MohamedObacz, JoannaChen, Cheng-HoOfiteru, Augustin M.Chen, FengOjha, Gunendra PrasadChen, I-Wen PeterOliva, RosarioChen, Jian-LianOnac, CananChen, Jian-ZhangOno, KatsushigeChen, JihuaOoi, Chien WeiChen, LukeOpiela, JolantaChen, MingtaoOprea, OvidiuChen, Shu-TingOrban, MathiasChen, Yong-SongOrbeci, CristinaCheng, JingcaiOrelle, CédricCherniha, Roman M.Ortiz-Medina, JosueCheshmedzhieva, DianaOrvin, KatiaChew, Jia WeiOsman, AhmedChinappi, MauroOssowicz, PaulaChmielewska, AleksandraOtahal, AlexanderCho, Hwa JinOthman, Siti HajarCho, KyungjinOvalle-Encinia, OscarCho, Young-JaePadan, EtanaChrcanovic, BrunoPai, Tzu–YiChristensen, Morten LykkegaardPalacio, Daniel A.Chu, XiangpingPalacio, LauraChuah, Chong YangPaloncýová, MarkétaChung, Neal Tai-ShungPandey, SadanandChung, Sheng-HengPandya, Anshul A.Chwojnowski, AndrzejPaneth, AgataCichero, ElenaPanneerselvam, BalamuruganCikurel, Eng. HaimPaolella, AndreaCiria, MiguelPaolone, AnnalisaCirillo, GiuseppePappalardo, FedericoComanescu, CezarPappas, George S.Cooper, Robin LewisParajo, Juan JoséCornelissen, EmileParchure, AnupCosta, JoanaPardo, FernandoCristian, PetcuParfenova, LyudmilaCroce, Anna CletaPark, Chul-hoCruz-Silva, RodolfoPark, IlhwanCui, Yan-HongPark, JaehoonCuli, JoaquimPark, Jin YongCundrle, IvanPark, Jin-SooCwiklik, LukaszPark, Jun-BeomCzaplewski, CezaryPark, KihoCzermak, PeterPark, Myoung JunCzernel, GrzegorzPark, Sang-HeeDadashi Firouzjaei, MostafaPark, Seong-JikD’Agostino, CarmineParkinson, BruceDammak, LasâadParthiban, AnburajanDamtie, Mekdimu MezemirParvasi, PayamDa’na, EnshirahPasini, MariaceciliaDanglot, LydiaPatel, DevenDanti, SerenaPatel, MalkeshkumarDarling, SethPathak, NirenkumarDarwish, MohamedPatki, NeilDas, RatulPatsios, SotirisDash, Santanu KumarPaulino, JoanaDavid, OanaPavli, FoteiniDe Angelis, Maria GraziaPeer, PetraDe Bruno, AlessandraPei, HaiyanDe Caroli, MonicaPenchev, HristoDe Craene, Johan-OwenPeng, HaoDe Falco, ElenaPeng, YuanDe Luca, GiorgioPerduca, MassimilianoDe Rosa, SalvatorePérez, GustavoDe Sequeira, César Augusto CorreiaPérez-Prior, M. T.De Simone, Salvatore GiovanniPergher, SibeleDe Souza, Paulo RicardoPergher, Sibele B.C.De Vries, Adrianus J.Petelska, AnetaDeana, CristianPetr, JanDement’ev, KonstantinPetriev, IliyaDemetrescu, IoanaPetuhov, OlegDenizot, BenoitPetukhov, AntonDerler, IsabellaPetukhov, Anton N.Desideri, EnricoPetukhov, Dmitriy I.Deuermeier, JonasPfisterer, SimonDeyev, IgorPickholz, Mõnica A.Dhar, GouravPiekutin, JaninaDi Bella, SantinaPiffoux, MaxDi Girolamo, RoccoPilipović, AnaDi Martino, GiuseppePilmane, MāraDi Palma, LucaPinho, LuísDi Profio, GianlucaPino, LidiaDi Scala, CoraliePismenskaya, NataliaDíaz López, OliverPivac, BrankoDikici, Betul AldemirPlisko, Tatiana V.Dinis, Maria Alzira PimentaPogrebnjak, AlexanderDiVerdi, JosephPokorny, MarekDixon, DavidPolańczyk, AndrzejDobrota, DanPolčic, PeterDolowy, KrzysztofPolitano, AntonioDołowy, KrzysztofPollice, AlfieriDong, DengpanPonomarev, Igor I.Dong, JuPontes, BrunoDonzelli, ElisabettaPontie, MaximeDordevic, DaniPopa, ElenaDoyon, NicolasPotkay, JosephDrenkova-Tuhtan, AsyaPoulsen, HanneDrescher, SimonPouraghaei Sevari, SevdaDrioli, EnricoPoveda Larrosa, Jose AntonioDroździk, MarekPoyatos, JoséDu, DeguoPradanos, PedroDu, HaishunPramanik, AvijitDudek, GabrielaPreta, GiulioDuerschmied, DanielPrieto Dapena, FranciscoDugar, Rohit P.Prudovsky, IgorDumee, LudovicPsarra, KaterinaDuranceau, Steven J.Punia, SnehDutournié, PatrickPurwidyantri, AgnesDutta, Ravi ChandraPutz, Ana MariaDzuba, Sergei AndreevichPyrc, MichałEfimova, Svetlana S.Qadir, DanialÉgée, StéphaneQanud, KhaledEhrentraut, Stefan FelixQazi, RazaEleonora, RussoQiu, GuangyuEljaddi, TarikQiu, MinghuiEljamal, OsamaQuevedo, Ivan R.Elkady, MarwaRaasakka, ArneElma, MuthiaRaceanu, MirceaEl-Newehy, MohamedRafiq, SikanderElSherbiny, Ibrahim M.A.Rahman, Mohammed M.Elsie, Parés-MatosRaikher, Yuriy L.Emmanouil, ChristinaRaj, NawinEscorihuela, JorgeRajabzadeh, SaeidEsmaili, MansooreRajca, MariolaEspuche, ElianeRamapuram, JayEum, KiwonRangelov, StanislavEwers, HelgeRanjan, AlokFabisiak, KazimierzRanjit, SabinaFalcon, DeboraRao, SanjeevFan, JiabingRätzke, KlausFarcasanu, Ileana C.Raudino, AntonioFarhan, MohammadRavichandran, KollarigowdaFarooq, SherR.B. Ola, AntoniusFashandi, HosseinReczek, LidiaFatyeyeva, KaterynaRein, AlanFaustino, VeraReinosa, Julián JiménezFedorov, Ruslan V.Renda, MarioFénelon, MathildeRicco, RaffaeleFeng, ShengjieRiva, GiuseppeFeng, ShilunRoatta, SilvestroFernandes, Maria HelenaRobles, AngelFernández-Tomé, María Del CarmenRocha, DjenisaFeroz, HasinRodrigo, LuisFerraz, Emanuela PradoRodrigues Reis, Cristiano E.Fievet, PatrickRodríguez-Gómez, Luis E.Filip, PetrRoedl, KevinFilippov, AnatolyRohner, PatrikFillafer, ChristianRona, RobertoFini, PaolaRostoker, GuyFiorenza, RobertoRoux De Balmann, HélèneFirdaous, LoubnaRovisco, AnaFirtat, BogdanRoy, SagarFisch, AdrianoRozalski, MarcinFlorán, BenjamínRózsenberszki, TamásFologea, DanielRuaro, BarbaraFontana, SimonaRubart, MichaelFontanesi, ClaudioRudnitskaya, AlisaFontàs, ClaudiaRuggero Neto, JoaoFrolova, Tatiana S.Ruiz, María OlgaFrontera, PatriziaRuiz-García, AlejandroFryczkowska, BeataRusso, FrancescaFu, HouqiangRuysschaert, Jean-MarieFuente, ElenaRyoji, ImaiFujii, YutakaRyskalin, LarisaFukuda, MakotoRyzhkov, Ilya I.Furukawa, KenjiRzymowska, JolantaFurukawa, MasashiSaavedra, Jorge H.Galindo, AntonioSadmani, A.H.M. AnwarGaman, Amelia MariaSadykov, VladislavGaman, Mihnea-AlexandruSaeed, SaqibGandasi, Nikhil R.Saeki, DaisukeGangadaran, PrakashSaez, Avelino EduardoGanguly, AnindyaSahu, SubinGao, LiSaid, Ahmed S.Garab, GyozoSaifutdinov, Bulat R.Garcia, AndreinaSaint-Pol, JulienGarcia-Bernabé, AbelSakaguchi, ToshikazuGarcía-Márquez, AlfonsoSakai, HiromiGarcia-Salaberri, Pablo A.Sakane, FumioGariglio, StefanoSakthivel, KogularasuGatto, IreneSalari, MarjanGaude, EdoardoSalaün, FabienGazdzicki, PawelSaleem, MuhammadGe, LiangSalit, Mohd SapuanGeise, Geoffrey M.Salman, MootazGeisler, RamsiaSalton, FrancescoGeorgieva, Elka R.Samhan-Arias, AlejandroGeorgin, JordanaSamsudin, Asep MuhamadGeorgopanos, ProkopiosSamsun, Remzi CanGhaffour, NoreddineSanctis, ShawnGhalkhani, MasoumehSanna, GavinoGhosal, KajalSanter, DavidGhosh, AbhishekSantiago, IbonGiacobbo, AlexandreSantoro, SergioGiagnorio, MattiaSantos, AdrianoGiangrieco, IvanaSantos, JerranGiannakas, ArisSapalidis, AndreasGica, NicolaeSarbu, AndreiGierszewska, MagdalenaSarris, Ioannis E.Gilassi, SinaSarti, Giulio C.Gilron, JackSasaki, NobuoGiorno, LidiettaSavard, ChristopheGlüsen, AndreasSawamura, Kentaro-ichiGoddard, AlanScarcia, PasqualeGoldmann, MichelScaturro, DalilaGonzales, Ralph RollySchcolnik-Cabrera, AlejandroGonzález, JoseScheidt, Holger A.Gonzalez, MiguelSchicker, KlausGonzález-Pérez, Daniel M.Schneck, EmanuelGorrín, Maite E. RiveraSchneckenburger, HerbertGosling, MartinSchneider, DirkGotzias, AnastasiosScholes, ColinGozálvez-Zafrilla, José MarcialSchroeder, GrzegorzGraça, CátiaSchulz, BurkhardGrahovac, JovanaSchwalbe, Ruth A.Granado Castro, María DoloresSdrobis, AnamariaGriepenburg, Julianne C.Seddighi, SadeghGrobner, GerhardŠegota, SuzanaGrueso, EliaSek, SlawomirGrushevenko, EvgeniiaSelyanchyn, RomanGrzegorzek, MartynaSemenycheva, Ludmila L.Guan, KechengSeong, Jong GeunGuaus, EsterSepahvand, SimaGüell, CarmeSequeira, César A. C.Guillamat-Prats, RaquelSergi, Pier NicolaGuillén, ElenaSetničková, KaterinaGüler, EnverSeyfaee, AhmadGumfekar, SarangSgreccia, EmanuelaGuminska, JolantaShafiquzzaman, MdGunendra Prasad, OjhaShahid, Muhammad KashifGuo, FeiShahkaramipour, NimaGuskov, AlbertShaik, Mohammed RafiGutierrez, LeonardoShankar, SarojiniGutierrez, Mar FernandezShao, LuGuzinski, MarcinShao, SenlinGuzmán, EduardoSharma, PrashantGuzzetta, FabrizioSharma, Priyanka R.Ha, Ngoc SanSheff, Joey G.Hadi, AlirezaShen, FeiHamawand, IhsanShen, QiuwanHannu, JariSher, FarooqHao, HongxiaShimokawa, NaofumiHaponiuk, JózefShishatskiy, SergeyHappel, ChristineShivange, AmolHaqqani, Arsalan S.Shrivastava, IndiraHardy, John G.Sibag, MarkHarja, MariaSiebenhofer, Matthäus S.Hartley, IanSiekierka, AnnaHasbullah, HasrinahSieni, ElisabettaHashimoto, TakashiSilva, AdrianoHawari, Alaa AlSilva, MónicaHay, SamSilva, ReneHayashi, MikioSilvers, RobertHe, ChengSimari, CataldoHe, ShaojianSimonescu, Claudia MariaHelenbrook, BrianSineshchekov, OlegHellmann, NadjaSingh, AjeetHelmi, ArashSingha, Nayan RanjanHendrik, BargelSingla, SaranshuHendrix, JelleSinha Ray, SaikatHenkensmeier, DirkSinner, EvaHernández, AgustínSisco, NIcholasHernández, Jose AlfredoSivakumar, ManiHeuer-Jungemann, AmelieSkoczko, IwonaHidalgo, Asuncion MariaSkouras, EugeneHii, Charles S.Ślusarczyk, CzesławHilarydoss, SharonSmarandache, AdrianaHingorani, RittuSmolka, PetrHirano-Iwata, AyumiSofue, TadashiHirota, YuichiroSokolova, Maria PetrovnaHlaibi, MiloudiSong, LianfaHnát, JaromírSong, ShuangHo, Chii-dongSoukup, KarelHo, JamesSousa, AngelaHo, Jia ShinSpartalis, MichaelHoffman, Dax AaronSperle, PhilippHolien, JessicaSpoială, AngelaHolt, Stephen A.Spychała, MarcinHolze, RudolfStamatialis, DimitriosHomaeigohar, ShahinStaszak, MaciejHomocianu, MihaelaStinghen, Andréa Emilia MarquesHoogenboom, BartStojmenović, MarijaHorbett, Thomas AStopic, SreckoHorn, HaraldStrzelewicz, AnnaHosseini, Seyed SaeidStyskalik, AlesHou, DianxunSu, FangyuanHou, JingweiSu, JincaiHsini, AbdelghaniSu, YajuanHu, AnmingSubedi, PramodHu, ChengzhiSuchaneck, GunnarHu, LeiqingSuckeveriene, RanHuang, LiangSuen, Jacky Y.Huang, Paul Li-HaoSugawara, AkiraHuang, Shu-HsienSugimura, Rio RyohichiHuet, EricSuk, PavelHumelnicu, DoinaSun, CaijunHwang, Jun YoungSun, ChuanyuIaquaniello, GaetanoSun, FeiyunIbaseta, NelsonSun, MengtaoIglic, AlesSunarso, JakaIhsanullah, IhsanullahSundarrajan, SubramanianIlia, GheorgheSung, Gun YongIliev, BoyanSviridov, DmitriImam, Syed SarimSwaraj, SufalImhof, PetraSymes, MarkImran Khan, MuhammadSysel, PetrIndiveri, CesareSzanto, IldikoInoishi, AtsushiSzczepanska-Sadowska, EwaIon-Ebrasu, DanielaSzczuka, AleksandraIorio, Ronald M.Sze, Lai LiIqbal, JavedSzekely, GyorgyIqbal, MuzaffarSzewczyk, AdamIqbal, ZafarSzulc, KarolinaIsakova, ElenaSzwast, MaciejIsmail, ManalSzymona, KarolinaIstrate, BogdanTabarkiewicz, JacekIvanets, AndreiTabraiz, ShamasIwata, TomoyukiTadros, MinaIzquierdo, MartaTaghvaei Nakhjiri, AliJacob, PaulTajiri, KazuyaJain, Bhawik KumarTakahashi, ShuntaroJameel, M.Takamuku, ShogoJanakiram, SaravananTakeda-Morishita, MarikoJanas, DawidTamašauskaitė-Tamašiūnaitė, LoretaJang, JaewonTan, Noel PeterJansen, Johannes Carolus (John)Tan, SongwenJasielec, Jerzy J.Tanaka, ShunsukeJasiński, MariuszTapajna, MilanJastrzab, RenataTarditi, Ana MariaJayakumar, ArunkumarTatullo, MarcoJena, Himanshu SekharTéllez, CarlosJeon, Jae-DeokTéllez-Valencia, AlfredoJeon, Ju-WonTemesvari, LeslyJeż-Walkowiak, JoannaTeng, Yu-NingJheng, Li-ChengTenopoulou, MargaritaJi, GuozhaoTeodosiu, CarmenJi, JiayuanThibault, JulesJiang, ChenxiaoTiraferri, AlbertoJiang, ShaohuaToikka, AlexanderJimenez, CeciliaTopuz, FuatJimenez-Jorquera, CeciliaTorices, Marcos FallanzaJo, ChangshinTorres, JaumeJobin, Marie-LiseTorricelli, MartinaJohnson, ShawnTortolini, CristinaJones, MarjorieToth, Andras JozsefJorge Guiomar, AntónioTotu, EugeniaJoshi, RakeshTow, EmilyJudge, Peter J.Tres, Marcus ViniciusJullok, NoraTricarico, DomenicoJunaedi, CholidTriolo, ClaudiaJung, Eun SangTronci, GiuseppeJůza, JosefTryggvason, GretarKabelac, StephanTryk, DonaldKabsch-Korbutowicz, MałgorzataTsai, Peichun AmyKahar, PrihardiTsehaye, Misgina TilahunKalas, WojciechTseng, Hui-hsinKallitsis, Joannis K.Tsvetkov, NikolaiKammakakam, IrshadTuran, JanKamyab, HesamTurczyn, RomanKanematsu, HideyukiUche Marcuello, Francisco JavierKang, CongBaoUhrmacher, Adelinde M.Kang, InpilUlbricht, MathiasKang, Moon-sungUllah, SanaKanjilal, BaishaliUmar, KhalidKanyukov, EgorUnterhuber, MatthiasKappert, EmielUpadhyay, ArunKarczewski, JakubUppulury, KarthikKardos, JuliannaUz, MetinKargar, FariborzVale, NunoKarimi, MohsenValenti, DanielaKarkhanehchi, HamedValenzuela, StellaKarn, Santosh Kr.Van Den Bogaart, GeertKassal, PetarVan Den Brink, Floris SKayo Santana, BarrosVan Der Bruggen, BartKazachenko, AlexandrVan Goethem, CédricKelarakis, AntoniosVancea, CosminKemperman, AntoineVandezande, PieterKerch, GarryVanek, OndrejKeshavarz Moraveji, MostafaVardhan, HarshKeun-hwan, OhVaresano, AlessioKhan, HilalVasicek, OndrejKhan, Mohammad EhtishamVasos, PaulKheawhom, SoorathepVassiliou, Alice GeorgiaKhorshidi, BehnamVázquez-Ibar, José LuisKikionis, StefanosVélez, MariselaKim, Ae RhanVelizarov, SvetlozarKim, Gue-HyunVeneziano, RemiKim, HankiVercelli, BarbaraKim, HyunukVermeulen, StevenKim, Ik-HwanVicente-Manzanares, MiguelKim, JeongVilé, GianvitoKim, Jin-chulVillalobos, Luis FranciscoKim, KyochanVinogradova, OlgaKim, Nam-HoonVirt, Ihor S.Kim, Sang MoonVishnyakov, AlekseyKim, SeungjuVochita, GabrielaKim, SoochongVoshkin, AndreyKim, YoungjinVujicic, MiloradKimura, MutsumiWales, DominicKingsbury, RyanWali, QamarKirichenko, KseniaWang, ChunyuKirsanov, DmitryWang, DingKitaguchi, TetsuyaWang, Hay-Yan J.Kizilova, NatalyaWang, JinliKlaysom, ChalidaWang, LeiKnauth, PhilippeWang, QiKnezevic, JelenaWang, Su-JaneKobrak, Mark N.Wasilewska, MonikaKoduru, JanardhanWatson, MichaelKołodyńska, DorotaWeihs, GustavoKolya, HaradhanWenten, I. GedeKolzunova, LidiiaWIbowo, Rachmat AdhiKong, LingxueWickham, ShelleyKonjevoda, PaškoWidiastuti, NurulKoók, LászlóWiedemann, DominikKorolkov, Ilya V.Wieland, D. C. FlorianKosel, JanezWilke, NataliaKöster, DariusWilm, MatthiasKostoglou, MargaritisWłodarczyk, BarbaraKoter, StanislawWłodarczyk, RenataKothandaraman, JotheeswariWojciechowska, MonikaKotulska, MalgorzataWołowicz, A.Koutavarapu, RavindranadhWołowicz, AnnaKovačević, DavorWong, Siu Hong DexterKowalewski, Mariusz P.Wood, Jeffery A.Kowalik-Klimczak, AnnaWorch, RemigiuszKragović, MilanWrotek, AugustKrasowska, MonikaWu, LiangKristl, MatjažWu, LiliKrivovichev, Gerasim VladimirovichWu, Sheng-NanKrystynik, PavelXia, LinglingKsepko, EwelinaXiao, KangKubo, Anna-LiisaXiaoyan, HuiKudłak, BłażejXie, JingxiuKujawa, JoannaXue, ShuangmeiKula, SławomirYadav, AnshulKulma, MagdalenaYadav, ShrutiKumabe, KazuhiroYadav, SudeshKumar, ManuYang, BoguangKumar, NavinYang, HsiharngKumar, RachanaYang, JieKumar, RameshYang, JixiangKumar, RanjithYasukawa, MasahiroKunli, GohYavari, MiladKurc, BeataYeh, Hsuan-HsienKuropka, PiotrYin, Ben HangKvitek, OndrejYoo, Dong JinKwon, ByungsuYoo, JeeyoungKyzas, GeorgeYoon, MinyoungKyzas, George Z.Yoshida, KentaroLa Deda, MassimoYounes, HammadLai, Chin WeiYoussry, MohamedLai, Gwo-SungYu, DaweiLamas-Samanamud, GisellaYu, FeiLameloise, Marie-LaureYuan, Chiun-JyeLammertink, Rob G.H.Zaffora, AndreaLanč, MarekZaid Alkilani, AhlamLancelin, Jean-marcŻak, KrzysztofLangowski, Horst-ChristianZakaria, Mohamed BarakatLansink-Hartgring, Annemieke OudeZakrocka, IzabelaLappan, UweZarejousheghani, MashaalahLapteva, MariaZarzycki, ArkadiuszLaradji, MohamedZdenek, SloukaLarchet, ChristianZdorovets, MaximLascu, AncaZec, NebojšaŁaskawiec, EdytaZenith, FedericoLasseuguette, ElsaZerbe, OliverLau, Woei JyeZhang, Daniel XinLe, Nguyen Quoc KhanhZhang, JianhuaLebiedzinska-Arciszewska, MagdalenaZhang, JiaweiLechiancole, AndreaZhang, KaiyueLee, AlbertZhang, LaibaoLee, Bor-ShiunnZhang, LiqunLee, ChangguZhang, MeijiaLee, ChulminZhang, MingjiLee, Eui-JongZhang, YongliangLee, IlkeunZhao, NanaLee, JaehanZhao, XiLee, Jyh-YeuanZheng, FanLee, Kew-HoZheng, LibingLee, Kueir-RarnZholkovskiy, EmiliyLee, SanghoZholobko, OksanaLee, Wei-ChiehZhou, JianLelis, MartynasZhu, ShanLenik, JoannaZienkiewicz, KrzysztofLeopold, Jane A.Zigon, PolonaLeskovac, Andreja R.Zivanovic, JasminaLeszczyńska-Sejda, KatarzynaZornoza, BeatrizLi, ChenZulkifli, MohammadLi, ChunleiZuo, JianLi, Hao



